# Supplementary material for: Surname affinity in Santiago, Chile: A network-based approach that uncovers urban segregation
Source: PLoS One. 2021 Jan 6;16(1):e0244372. doi: 10.1371/journal.pone.0244372 (PMC7787389; doi:10.1371/journal.pone.0244372)
Supplement: S1 Table — Selected surnames are those with the highest degrees within each community. (PDF) [file pone.0244372.s001.pdf]

S1 Table. Top-10 surnames per community in the paternal-maternal surname affinity network. Selected surnames are those with the highest degrees within each community.

CID	SES_{mean}	SES_{sd}	Top-10 surnames
C0	82.1	7.3	Awad, Jadue, Hasbun, Manzur, Nazar, Ananias, Alamo, Zaror, Haddad, Hirmas.
C1	55.8	8.0	Nicolich, Savich, Aristich, Pantich, Arestich, California, Caldera, Aristides, Ilich.
C2	42.9	3.5	Carilao, Lienlaf, Quilapan, Curinao, Pitriqueo, Rucal, Colipe, Mulato, Nahuelpan, Ancaten.
C3	76.3	5.1	Lee, Kim, Park, Choi, Chung, Hong, Chen, Yang, Sung, Jung.
C4	40.2	3.6	Painen, Curihuinca, Colihuinca, Cona, Cayuleo, Quintriqueo, Llancaleo, Collio, Huente, Huircapan.
C5	80.4	9.2	Ergas, Errazuriz, Aspillaga, Camhi, Cohen, Irarrazaval, Schmidt, Ventura, Laso, Court.
C6	75.0	8.1	Martorell, Espinace, Feliu, Hadad, Hahn, Neyra, Contador, Heresmann, Raveau, Solari.
C7	79.7	9.2	Edwards, Zanartu, Monckeberg, Lyon, Alessandri, Subercaseaux, Besa, Braun, Mackenna, Vial.
C8	68.7	8.4	Reuque, Becker, Bassi, Mezzano, Leniz, Lopetegui, Zevallos, Soler, Astorquiza, Bas.
