# Supplementary material for: Surname affinity in Santiago, Chile: A network-based approach that uncovers urban segregation
Source: PLoS One. 2021 Jan 6;16(1):e0244372. doi: 10.1371/journal.pone.0244372 (PMC7787389; doi:10.1371/journal.pone.0244372)
Supplement: S2 Table — Prominent surnames are those frequent in an area but infrequent in the other areas. Frequent and infrequent lists of surnames were computed using the top-500 lists of salient surnames in each area. (PDF) [file pone.0244372.s002.pdf]

S2 Table. Top-10 surnames per community detected in the isonymy surname affinity network. Prominent surnames are those frequent in an area but infrequent in the other areas. Frequent and infrequent lists of surnames were computed using the top-500 lists of salient surnames in each area.

CID	SES_{mean}	SES_{sd}	Top-10 surnames
1 (yellow)	33.2	21.2	Cruces, Grandon, Arriaza, Millar, Moscoso, Quilodran, Bascur, Saravia, Viveros, Zarate.
2 (blue)	92.7	9.6	Larrain, Cruzat, Iturriaga, Labarca, Ruiz-Tagle, Velasco, Edwards, Labbe Garcia-Huidobro.
3 (red)	56.1	23.1	Quevedo, Jeria, Moyano, Polanco, Melendez, Solar, Ubilla, Escudero, Pezoa, Montes.
4 (green)	37.4	23.2	Canete, Moreira, Corrales, Narvaez, Paz, Escobedo, Farfan, Sotelo, Tudela, Valdenegro.
