# Supplementary material for: Surname affinity in Santiago, Chile: A network-based approach that uncovers urban segregation
Source: PLoS One. 2021 Jan 6;16(1):e0244372. doi: 10.1371/journal.pone.0244372 (PMC7787389; doi:10.1371/journal.pone.0244372)
Supplement: Attachment — Submitted filename: response_to_reviewers.pdf [file pone.0244372.s003.pdf]

RESPONSE TO REVIEWERS

Dear Editors and Reviewers:

In this revised version of our manuscript we have accurately taken care of all the questions asked by the reviewers, as thoroughly described in the remainder of this letter.
We appreciated all the insightful comments we received in response to our submission. These useful suggestions and observations helped us improve and strengthen our manuscript. Please, find below the original comments by the Editor and the Anonymous Reviewers, together with a thorough explanation of how we addressed them. In order to support the revision process, we have also highlighted in yellow all the modified paragraphs in the revised manuscript.

Thank you in advance for your time and consideration.

Yours faithfully,

Naim Bro and Marcelo Mendoza

Journal requirements

Comment:
Please ensure that your manuscript meets PLOS ONE's style requirements, including those for file naming.

Reply: Thanks for the observation. Now we provide a revised version of the paper that follows all the guidelines indicated by the editor. Please see the detailed actions below.

Action:

- Major sections titles were updated changing the font size to 18pt using bold fonts; level 2 headings were written using 16pt bf, .
- Figures are cited as "Fig 1", "Fig 2", etc. We also reorganized the paper figures, including them immediately after their first mention in the text. We included Figure 2 in the paper's body. In the previous version of the paper this figure was included in the appendix. We did this change as Figure 2 was connected to the text in Section of results.

Comment:
We note that you have stated that you will provide repository information for your data at acceptance. Should your manuscript be accepted for publication, we will hold it until you provide the

relevant accession numbers or DOIs necessary to access your data. If you wish to make changes to your Data Availability statement, please describe these changes in your cover letter and we will update your Data Availability statement to reflect the information you provide.

Reply: Thanks for this observation. In the current version of the paper, we comment on how and where we share the data that support the study. Please see the detailed list of actions below to fulfill this requirement.

Action (revised l. 64-65):

We release the data and networks used in the paper in figshare. Please see the collection in: https://doi.org/10.6084/m9.figshare.c.5230835.v1. The collection includes 5 files related to the isonymy network and 1 file for the maternal-paternal surname network. The isonymy network data provided in this collection covers the files areas.csv (manzana-areas), coords.csv (id, coordinates (latitude, longitude), manzana, area), ED.npy (numpy array of Euclidean distances used to create the network), santiago.csv (id, label, modclass, and many network features), and santiago.gephi (gephi file of the network). The paternal-maternal surname network provides one file named surnames.gml, which comprises the whole network. We include a reference for the collection in the paper in lines 64-65, where the data is introduced.

Comment:
Please include captions for your Supporting Information files at the end of your manuscript, and update any in-text citations to match accordingly.

Reply: Thanks for the observation. In this new version we replaced the appendix with two supporting information files. We declared the supporting information at the end of the document's body. We also modified the reference to the appendix by the one to the supporting information files.

Action:

Please see the Supporting information section at the last part of the paper. We included two tables in pdf files to fulfill with the journal requirement. We also modified the reference to the appendix by the one to the supporting information files.

Comment:
We note that [Figure(s) 2 and 5] in your submission contain [map/satellite] images which may be copyrighted. All PLOS content is published under the Creative Commons Attribution License (CC BY 4.0), which means that the manuscript, images, and Supporting Information files will be freely available online, and any third party is permitted to access, download, copy, distribute, and use these materials in any way, even commercially, with proper attribution. For these reasons,

we cannot publish previously copyrighted maps or satellite images created using proprietary data, such as Google software (Google Maps, Street View, and Earth). For more information, see our copyright guidelines: http://journals.plos.org/plosone/s/licenses-and-copyright.

Reply: Thanks for this observation. We completely remade these figures to comply with the copyright agreements indicated by the publisher. To achieve this, we generate the images using a layer of polygons on a metropolitan region's raster layer. The polygon layer was generated by us in qgis so that the property of this layer is ours. Then, the raster layer was removed. The images were remade in order to display the information across communes. Please see the details of these actions in the box below.

Action (revised Fig 5 and Fig 6b):

The nine plots in Fig 5 (old Fig 2) were created using a layer of polygons representing the territorial division of the Metropolitan region. The map is shown segmented by communes. The colors indicate the presence of the community in each commune. Only communes with a presence of at least 10% of the indicated community have been colored. The more intense colors indicate a higher presence of the community in that area. In the case of Fig 6 (old Fig 5), we remade the fig b using the colors that indicate each of the four communities detected using the isonymy network.

Comment:

Please include your amended statements within your cover letter; we will change the online submission form on your behalf. Please expand the acronyms "MM ANID PIA/APOYO AFB" and "MM ANID FONDECYT" (as indicated in your financial disclosure) so that it states the name of your funders in full. This information should be included in your cover letter; we will change the online submission form on your behalf. *We note that you have provided funding information that is not currently declared in your Funding Statement. However, funding information should not appear in the Acknowledgments section or other areas of your manuscript. We will only publish funding information present in the Funding Statement section of the online submission form. Please remove any funding-related text from the manuscript and let us know how you would like to update your Funding Statement. Currently, your Funding Statement reads as follows: "MM ANID PIA/APOYO AFB180002 https://www.anid.cl NO MM ANID FONDECYT grant 1200211 https://www.anid.cl NO"*

Please include your amended statements within your cover letter; we will change the online submission form on your behalf. Please expand the acronyms "MM ANID PIA/APOYO AFB" and "MM ANID FONDECYT" (as indicated in your financial disclosure) so that it states the name of your funders in full. This information should be included in your cover letter; we will change the online submission form on your behalf.

Action (revised funding statement):

- We removed the funding statement from the acks section.
- Amended funding statements are:
Naim Bro and Marcelo Mendoza acknowledge funding support from the Millennium Institute for Foundational Research on Data.
Marcelo Mendoza was funded by the National Agency of Research and Development (ANID) grants Programa de Investigación Asociativa (PIA) AFB180002 and Fondo Nacional de Desarrollo Científico y Tecnológico (FONDECYT) 1200211.

Editor Comments

Comment 1:
Bro and Mendoza do a great job of describing the surname relationships among the population of Santiago, the links with urban distribution and segregation, and with SES.

Reply: We thank the editor for the positive feedback.

*Still, the paper falls short on the interpretation of its findings, and, while the conclusion hints to future analyses, some are needed in this paper. Just two examples: * Why two Mapuche paternal-maternal surname clusters?*

Reply: Thanks for this valuable observation. We expanded the contextual and interpretative components of the paper to make the findings more relevant to the public discussion.

Action (revised section results, paternal-maternal surname network (l. 164-168)):

We added some comments about the partition of Mapuche surnames into two groups. We provide some potential reasons that explain this split.

---

* Why the rich have more diverse surnames? It seems that the upper class contains different ethnic groups, and I suggest that the non-Chilean reader needs some context on how the richer Santiagoans have such diverse backgrounds. It could be contrasted with the also surname-diverse upper SES class in Spain, which forced diversity by seeking to differentiate their surnames from those of the commoners (Collado et al., investigaciones económicas. vol. XXXII (3), 2008, 259-287)

Reply: Thank you for this observation. We have added a paragraph adding the suggested reference, comparing Chile to Spain, and positing two reasons why the elites possess rare last names.

✎ Action (revised section results, isonymy network (l. 235-244)):

We added a paragraph including the suggested reference (Collado et al), comparing Chile to Spain, and formulating two reasons why the Chilean elites may have rarer last names than most people.

Comment 2:

A few lesser issues:
** l. 60: How was SES transformed into a 0-100 range? Please provide the equation used*

✎ Action (revised l 60-62):

In lines 60-62, we added the formula to transform SES into a 0-100 range, as requested.

** l. 171-172: please add a table or graph with the mean and sd of the SES values per surname cluster*

✎ Action (revised S1 Table and S2 Table):

We included mean, and standard deviation of the SES for each community detected in our analysis. These results are shown in the supporting information section, in Tables 1 and 2.

** l. 174-181 and Fig. 9: I was wondering whether 1830, which is the start point of the political analysis, precedes the arrival of some migrant groups, and thus may be biased against them*

✎ Action (revised l. 184-187):

We added a caveat in lines 184-187, regarding Fig. 9, indicating the non-Hispanic last names were probably underrepresented in the nineteenth-century Chilean congress, prior to the arrival of a large number of European or Middle Eastern immigrants.

Comment 3:
English:
** l. 84 sensitivity → sensitive*
** l. 92 this type's networks → the networks of this type*
** l. 97 similar number → similar numbers*
** l. 99 choose → chose*

Reply: Thanks for these observations.

Action:

* l. 84 sensitivity → sensitive; Done
* l. 92 this type's networks → the networks of this type; Done
* l. 97 similar number → similar numbers; Done
* l. 99 choose → chose; Done

Reviewer #1

Comment:
I think this is a well written and well presented paper. Data, methods and analysis are clearly set out and results are nicely presented. I only have one comment regarding Figure 2 - I assumed this was based on a Kernel Density Estimation, the results of which can be highly variable depending on the input parameters. I think these need to be consistent across the maps (I'm not sure if this is presently the case) and articulated. The color ramp is also problematic since it can exaggerate some parts of the distribution and is not color blind friendly. Take a look at colorbrewer.org for alternatives. You might also consider a more straightforward representation such as cell counts to show the same information.

Reply: We thank the reviewer for the positive feedback. We completely remade Fig 2 (now Fig 5) to follow the recommendation provided by the reviewer. To achieve this, we generate the images using a layer of polygons on a Metropolitan region's raster layer. The polygon layer was generated by us in qgis. Then, the raster layer was removed. The images were remade in order to display the information across communes. Please see the details of these actions in the box below

Action (revised Fig 5):

The nine plots in Fig 5 (old Fig 2) were created using a layer of polygons representing the territorial division of the Metropolitan region. The map is shown segmented by communes. The colors indicate the presence of the community in each commune. Only communes with a presence of at least 10% of the indicated community have been colored. The more intense colors indicate a higher presence of the community in that area. In this new version of these figures, the information between the images used to display the colors is comparable since we use a threshold to color the communes related to the community's presence in the area. This makes it possible to visualize that communities 6 and 8 have a weak territorial presence, unlike the rest that show a clear territorial presence.
